# Draft genome sequence of *Bacillus safensis* strain SMT-MG31 isolated from mangrove sediment of Bangladesh

**DOI:** 10.1128/mra.00469-26

**Published:** 2026-07-28

**Authors:** Mohammad Azizul Hoque, Md. Sheikh Emon, Ferose Kabir, Md. Majibur Rahman, S. M. Tanjil Shah

**Affiliations:** 1 Department of Microbiology, University of Dhaka 95324, Dhaka, Bangladesh; Rochester Institute of Technology, Rochester, New York, USA

**Keywords:** *Bacillus safensis*, draft genome, bioactive compounds, mangrove

## Abstract

*Bacillus safensis*, a microorganism, is known for its environmental resilience and biotechnological relevance. Here, we report the draft genome sequence of strain SMT-MG31, isolated from mangrove sediment in Bangladesh, to support investigations into bioactive compound-producing bacteria from marine-associated ecosystems.

## ANNOUNCEMENT

*Bacillus safensis* is a gram-positive, rod-shaped, endospore-forming mesophilic bacterium originally described from a spacecraft assembly facility (1). Since its initial characterization, it has been detected in diverse environments, including soil, rhizosphere, food-associated substrates, and other stress-prone habitats (2, 3). Its ecological adaptability and enzymatic diversity highlight its biotechnological potential (4).

Mangrove ecosystems are dynamic coastal environments characterized by salinity fluctuations, tidal influence, and high organic matter content (5). These selective pressures favor metabolically versatile microorganisms capable of producing bioactive secondary metabolites (6). As part of a study aimed at identifying novel bioactive compound-producing bacteria from mangrove sediment, strain SMT-MG31 was isolated from sediment collected in 2022 at Dublar Char, Bagerhat, Bangladesh.

For isolation, the raw sediment sample was boiled at 100°C to eliminate non-spore-forming microorganisms, followed by inoculation onto actinomycetes isolation agar medium. For whole-genome sequencing, genomic DNA was extracted using the DNeasy Blood & Tissue Kit from an overnight culture of the isolate grown in tryptic soy broth at 37°C. Library preparation was performed at the icddr,b Genomics Center (iGC) using the Illumina DNA Prep Kit, and sequencing was conducted on an Illumina MiSeq System generating 2 × 250 bp paired-end reads. No shearing or size selection of DNA was performed prior to sequencing. Raw reads were assessed for quality using FastQC (Galaxy version 0.47) and quality filtered and trimmed with Trimmomatic (Galaxy version 0.36.6). High-quality reads were assembled *de novo* using SPAdes (Galaxy version 4.2.0), and assembly quality metrics were evaluated with QUAST (Galaxy version 5.3.0). Genome completeness and contamination were estimated using CheckM (version 1.2.4), and species-level identification was confirmed by average nucleotide identity (ANI) analysis using FastANI (version 1.3) (7). The genome was annotated using the NCBI Prokaryotic Genome Annotation Pipeline (PGAP, software revision 6.10) (8). Default parameters were used for all software.

The draft genome of *Bacillus safensis* strain SMT-MG31 consists of 3,682,742 bp with a GC content of 41.68%. *De novo* assembly generated 40 contigs (≥200 bp), with the largest contig measuring 980,049 bp, an N50 of 527,784 bp, and an N90 of 320,005 bp (L50 = 3, L90 = 6), with no ambiguous bases detected. Sequencing produced 958,810 paired-end reads, resulting in an average genome coverage of 103.6×. Genome completeness and contamination, assessed using CheckM, were 99.59% and 0.21%, respectively, with no detectable strain heterogeneity. Species-level identification was confirmed using FastANI, which showed 97.21% ANI with the type strain *Bacillus safensis* FO-36b (GenBank accession: ASJD00000000), confirming the assignment of strain SMT-MG31 to *B. safensis*. Genome annotation using the NCBI PGAP (revision 6.10) predicted 3,798 genes, including 3,655 protein-coding genes, 114 RNA genes (rRNA, tRNA, and ncRNA), and 29 pseudogenes, demonstrating that this is a high-quality, near-complete genome. Genome mining using antiSMASH identified 11 putative biosynthetic gene clusters, including those associated with bacillibactin, bacilysin, plantazolicin, lichenysin, fengycin, schizokinen, and zwittermicin A biosynthesis, highlighting the strain’s biotechnological potential.

## Data Availability

This whole-genome sequence has been deposited in DDBJ/ENA/GenBank with accession number JBUQDM000000000 under BioProject accession number PRJNA1420374 and BioSample accession number SAMN55215617. The accession number of the publicly available raw data of the SMT-MG31 genome sequence is SRR38139914.
